# A Comprehensive Survey of Genomic Alterations in Gastric Cancer Reveals Recurrent Neoantigens as Potential Therapeutic Targets

**DOI:** 10.1155/2019/2183510

**Published:** 2019-11-05

**Authors:** Chao Chen, Qiming Zhou, Riping Wu, Bo Li, Qiang Chen, Xiuqing Zhang, Chunmei Shi

**Affiliations:** ^1^BGI Education Center, University of Chinese Academy of Sciences, Shenzhen 518083, China; ^2^BGI-Shenzhen, Shenzhen 518083, China; ^3^BGI-GenoImmune, Wuhan 430079, China; ^4^Hospital of Shenzhen Nanshan District, Shenzhen 518052, China; ^5^Fujian Medical University Union Hospital, Fuzhou 350001, China; ^6^Fujian Provincial Key Laboratory of Translational Cancer Medicine, Fuzhou 350004, China; ^7^Fujian Medical University Stem Cell Research Institute, Fuzhou 350004, China

## Abstract

Immunotherapy directed against cancer-specific neoantigens derived from non-silent mutants is a promising individualized strategy for cancer treatment. Neoantigens shared across patients could be used as a public resource for developing T cell-based therapy. To identify potential public neoantigens for therapy in gastric cancer (GC), 74 GC patients were enrolled in this study. Combined with the TCGA cohort and other published studies, whole exome sequencing data from 942 GC patients were used to detect somatic mutations and predict neoantigens shared by GC patients. The mutations pattern between our study and the TCGA cohort is comparable, and C > T is the most common substitution. The number of neoantigens was significantly higher in older patients (age ≥60) compared to younger patients (age <60), both in this study and the TCGA cohort. Recurrent neoantigens were found in eight genes (*TP53*, *PIK3CA*, *PGM5*, *ERBB3*, *C6*, *TRIM49C*, *OR4C16*, and *KRAS*) in this study. The neoantigen-associated mutations *PIK3CA* (p.H1047R) and *TP53* (p.R175H) are common across several cancer types, indicating their potential usage. Overall, our study illustrates a comprehensive genomic landscape of GC and provides the recurrent neoantigens to facilitate further immunotherapy.

## 1. Introduction

Gastric cancer (GC) is the leading cause of cancer-related death around the world and is particularly prevalent in east Asian countries [1, 2]. Chemotherapy and radiotherapy are the traditional treatment methods for GC. Despite the enhancement in eradication of *Helicobacter pylori* and early cancer screening, the 5-year overall survival rate for GC worldwide is still below 30% [3].

With the development of biologics, immunotherapy has revolutionized oncology by targeting the host immune system. There are many ways of immunotherapy, such as immune checkpoint inhibitors (ICIs), cancer vaccine, and mutant-antigen-specific tumor-infiltrating lymphocytes (TILs) [1, 4–7]. Recent studies have highlighted the role of tumor-specific neoantigens in cancer immunotherapy, promoting personalized vaccines and cell therapies based on cancer somatic mutations [8, 9]. Several studies have used neoantigens as targets in T cell-based immunotherapy for treatment of melanoma [10, 11], malignant gliomas [12], ovarian cancer [13], and breast cancer [4]. Although neoantigen-based immunotherapy had been used in many cancers, they have not been used in GC as far as we know.

The emergence of next-generation sequencing technology has rapidly expanded our understanding of the genetic basis of GC, and many studies have provided useful cross-validation information [14–20]. However, most of these studies focused on the mechanism of gene mutation and tumorigenesis but did not involve the neoantigen landscape of GC, and the sample size of a single study was limited. Zhou and colleagues have analyzed the neoantigens in 32 patients with GC. However, due to the limitations of sample size and detection methods, the study did not find neoantigens shared among GC patients [21]. Therefore, in order to promote the immunotherapy of GC based on neoantigens, we combined 74 samples of this study with published sequencing data to obtain 942 samples and carried out the largest study of the neoantigen landscape of GC so far.

## 2. Materials and Methods

### 2.1. Genomic Data of GC

This study was approved by the Ethical Committee of the Union Medical College Hospital Affiliated of Fujian Medical University and carried out in accordance with the approved guidelines. All patients signed informed consent before admission. In total, samples of cancer and adjacent normal tissues from 75 cases with clinical pathological information were provided, and 74 of them (hereafter referred to as Fujian cohort) passed the quality control for library construction and sequencing (Table 1). Besides, all somatic mutations, including single nucleotide substitutions and short insertion/deletions (indels), from the most recent publications (Table 1 and Supplementary Tables [Supplementary-material supplementary-material-1]-[Supplementary-material supplementary-material-1]), representing the other six geographically different research cohorts were downloaded, and the mutation data of 942 patients were eventually obtained for integrated analysis.

### 2.2. Pipeline for Somatic Mutation Analysis and Neoantigen Prediction

The sequencing data of Fujian cohort were processed with SOAPnuke [22] and mapped to human hg19 reference using Edico (http://edicogenome.com/dragen-bioit-platform), and then MuTect [23] and Varscan2 [24] were used to detect the somatic snvs and indels, respectively. Mutations with frequency >0.5% in common mutation databases (1,000 genomes database, Exome Sequencing Project 6500 database, dbSNP database, and Exome Aggregation Consortium database) were filtered. The final mutations were annotated with ANNOVAR [25] and transformed to MAF format using Maftools [26] for further statistical analysis and visualization. Some mutation sites were selected for mass spectrometry verification, and the validation rate was 94% [27].

The HLA genotyping of the Fujian cohort was processed using Polysolver [28], with the previous mapping bam file as input. Data of 408 TCGA samples containing HLA information were downloaded from the dbGAP database. Then, nonsilent snvs in Fujian (*n* = 74) and TCGA cohorts (*n* = 408) were used to predict neoantigens by NetMHC [29], NetMHCpan [30], PickPocket [31], PSSMHCpan [32], and SMM [33]. Peptides need to satisfy the following three criteria: (1) length between 8 and 11 mers; (2) affinity IC50 < 500 nM in at least two tools; (3) and the affinity score in mutation-type (MT) peptide is less than that in wild-type (WT) peptide.

### 2.3. Statistical Analysis

Statistical analyses were carried out by R studio, and the significance was determined by the Wilcoxon rank sum test or Fisher's exact test, when appropriate. When *p* < 0.05, it is defined as significant.

## 3. Results

### 3.1. Integrated Mutation Landscape of GC Patients

**Figure 1:**
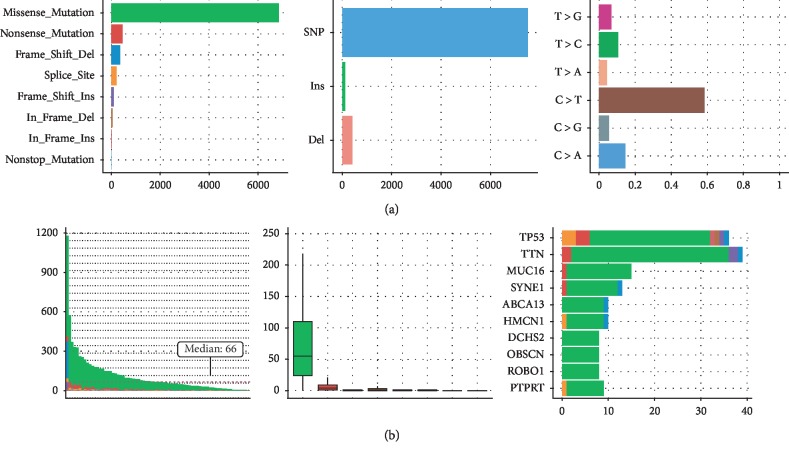
Mutation landscape in Fujian cohort. (a) From left to right, counts of each variant classification, counts of each variant type, and counts of each SNV class are presented. (b) From left to right, variants number per sample, variant classification, and top 10 significantly mutated genes are presented.

The mutations of GC in Fujian cohort were identified, and the landscape is shown in Figure 1. Totally, there are 10,607 somatic snvs and 511 somatic indels (insertions and deletions), with a median of 66 mutations across samples. C > T transition is the main type of mutations, consistent with TCGA and previous reports [19, 34]. The nonsilent snvs, including 6,857 missense mutations, 463 nonsense mutations, 9 nonstop mutations, and 212 mutations in the splice site were used to predict neoantigens.

For integrated analysis of the 942 GC samples, *TP53*, *TTN*, *MUC16*, *LRP1B*, *SYNE1*, and *CSMD3* are the most frequently mutated genes ([Supplementary-material supplementary-material-1]). Several other cancer driver genes, such as *ARID1A*, *FAT4*, and *PIK3CA*, were also found to mutated in more than 10% of GC samples. Interestingly, we found five genes were mutated exclusively with *TP53*, including *CDH1*, *KMT2D*, *RYR1*, *PIK3CA,* and *ARID1A* (Fisher's exact test, *P* < 0.05, [Supplementary-material supplementary-material-1]). The inverse relationship between *ARID1A* and *TP53* was reported previously [35], which indicates different cancer drive mechanisms. *CDH1*, *KMT2D*, *RYR1*, and *PIK3CA* were also concordantly mutated with *ARID1A* (Fisher's exact test, *P* < 0.05, [Supplementary-material supplementary-material-1]), suggesting that the carcinogenic mechanism of GC samples that carry mutations in these genes is similar but different from that of samples carrying *TP53* mutation.

### 3.2. Neoantigen Profiling of GC Patients

To explore the neoantigen profiling of GC, we predict the neoantigens for Fujian cohort and TCGA cohort using the nonsilent point mutations, separately. The number of neoantigens in Fujian cohort ranged from 0 to 753, with a median of 76. A total of 408 GC samples from TCGA were used to predict neoantigens. The number of neoantigens ranged from 2 to 15268, with a median of 193. The reason why the number of neoantigens in TCGA is more than that in Fujian cohort may be that the samples contain more mutations.

**Figure 2:**
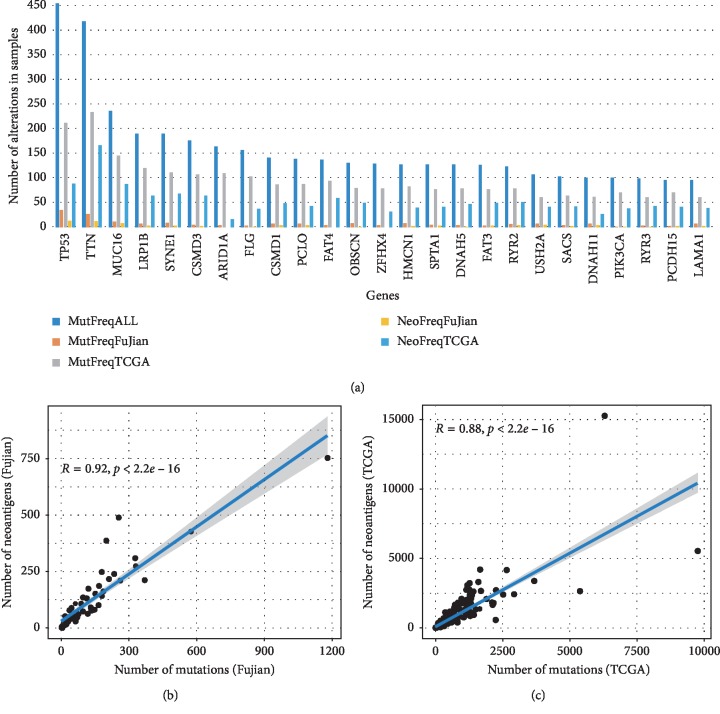
The overall number of somatic mutations and neoantigens in Fujian and TCGA cohorts. (a) The frequency of mutated genes and their neoantigens in Fujian and TCGA and all GC samples in this study (*n* = 942). The fitted curve between the number of nonsilent somatic mutations and neoantigens in Fujian cohort (b), *R*^2^ = 0.92, and TCGA cohort (c), *R*^2^ = 0.88, respectively.

Next, we want to know whether the significantly mutated genes (SMGs) also carry more neoantigens. The results show that the SMGs can produce more neoantigens (Figure 2(a)), such as *TP53*, *TTN*, *MUC16*, and *ARID1A*, which indicates that these genes may carry potential tumor targeting sites. *TP53*, which had non-silent mutations in 212 (48.5%) TCGA samples and 35 (47.3%) Fujian samples, respectively, produced the largest number of neoantigens in the two cohorts, with 89 and 13 samples carried neoantigens, respectively. *MUC16* is a gene that is positively related to the mutation load of tumors and can encode the tumor antigen CA-125. It is believed that the mutation of *MUC16* is associated with better prognosis [3]. In the two cohorts of this study, the gene *MUC16* also produced many neoantigens.

We further compared the correlation between the number of neoantigens and non-silent mutations in TCGA and Fujian cohorts. The results showed that, in both cohorts, the number of neoantigens was significantly correlated with the number of non-silent mutations, and the Spearman correlation coefficients were 0.92 (*P* < 0.01) and 0.88 (*P* < 0.01), respectively (Figures 2(b) and 2(c)).

### 3.3. Comparison of Neoantigens in Different Subtypes and Cohorts of GC

**Figure 3:**
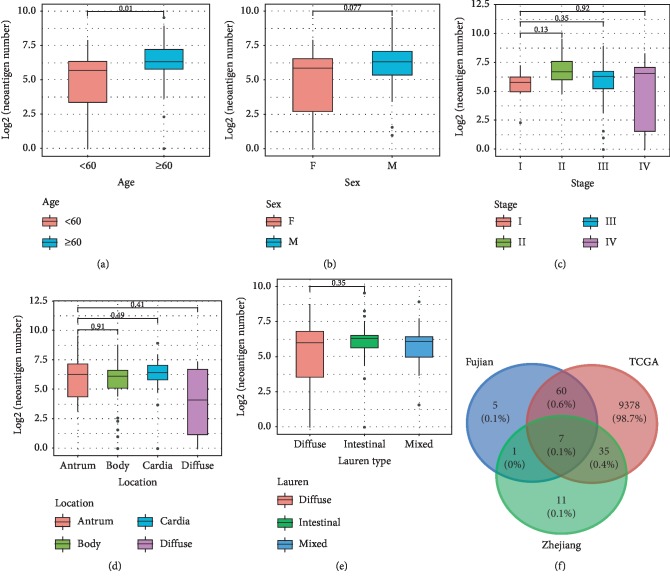
The comparation of neoantigens between different subgroups and cohorts: (a) between age ≧60 and age <60 groups; (b) between female and male groups; (c) between different stages; (d) between different locations; (e) between different Lauren types; (f) between different cohorts.

We then grouped the samples according to age, sex, Lauren type, stage, and location of occurrence, and counted the differences in the number of neoantigens between different subgroups. We found that patients older than 60 carried more neoantigens than patients younger than 60 (Wilcoxon rank sum test, *P*=0.01). Males tended to carry more neoantigens than females, but not statistically significant (Wilcoxon rank sum test, *P*=0.077, Figures 3(a) and (b)). There was no significant difference in the neoantigen load between different Lauren types, stages, and different locations (Figures 3(c)–3(e)). The same trend was observed in TCGA samples.

Zhou et al. found that 54 genes could produce neoantigens in at least three samples in the Zhejiang cohort [21]. In the Fujian and TCGA cohorts, 2,855 and 15,791 genes carrying neoantigens were detected, and 73 and 9480 genes were found in at least three samples, respectively. Comparing the three cohorts, we found that most of the genes could be covered by the TCGA and Fujian cohorts (Figure 3(f)). At the neoantigen level, no neoantigens shared among GC patients were found in Zhejiang cohort. Two and 486 neoantigens appeared in at least two samples in Fujian and TCGA, respectively. For the reason that the Zhejiang cohort did not find a common neoantigen sequence among GC samples, we speculate that it may be because the size of Zhejiang cohort is too small and they only use NetMHCpan software to predict neoantigens in their research, but the software can only predict a relatively small set of HLA class I alleles [30, 32], so there are some limitations in the prediction of neoantigens.

### 3.4. Neoantigens Shared among GC Patients

A total of 74,864 neoantigens were detected in Fujian and TCGA samples, of which 549 were found in at least 2 samples (a total of 61 neoantigens were shared between TCGA and Fujian cohorts). The top eight neoantigen associated genes were *PGM5*, *TP53*, *TRIM49C*, *PIK3CA*, *ERBB3*, *C6*, *OR4C16*, and *KRAS*, respectively (Table 2). If we consider these neoantigens as a panel, it can cover about 15.8% of the total 482 samples. At present, 10–20 cancer-specific neoantigens are usually synthesized in vitro and used in T cell immunotherapy [11]. Since these neoantigens can cover a certain proportion of GC population, we believe that these recurrent neoantigens of GC have potential clinical application value.

We found that the mutations corresponding to high frequency neoantigens in Fujian and TCGA samples were mostly high frequently mutated sites in all 942 GC samples and TCGA pan-cancer (Table 2, Freq1, Freq2, and Freq3, respectively). Although not all samples can obtain HLA information, we believe that these neoantigens are also potential tumor-specific neoantigens in other GC samples.

### 3.5. Hotspot Mutation-Related Neoantigens That May Be a Potential Source of Immunotherapy Target in GC and Pan-Cancer

In order to further study the potential significance of these high frequency neoantigens, we focused our attention on the mutation R175H (*TP53*), H1047R (*PIK3CA*), and G12D (*KRAS*) because these sites can not only produce recurrent neoantigens (frequency of more than 4 occurrences in 482 samples) but also have high mutation frequencies in the TCGA pan-cancer cohort.

**Figure 4:**
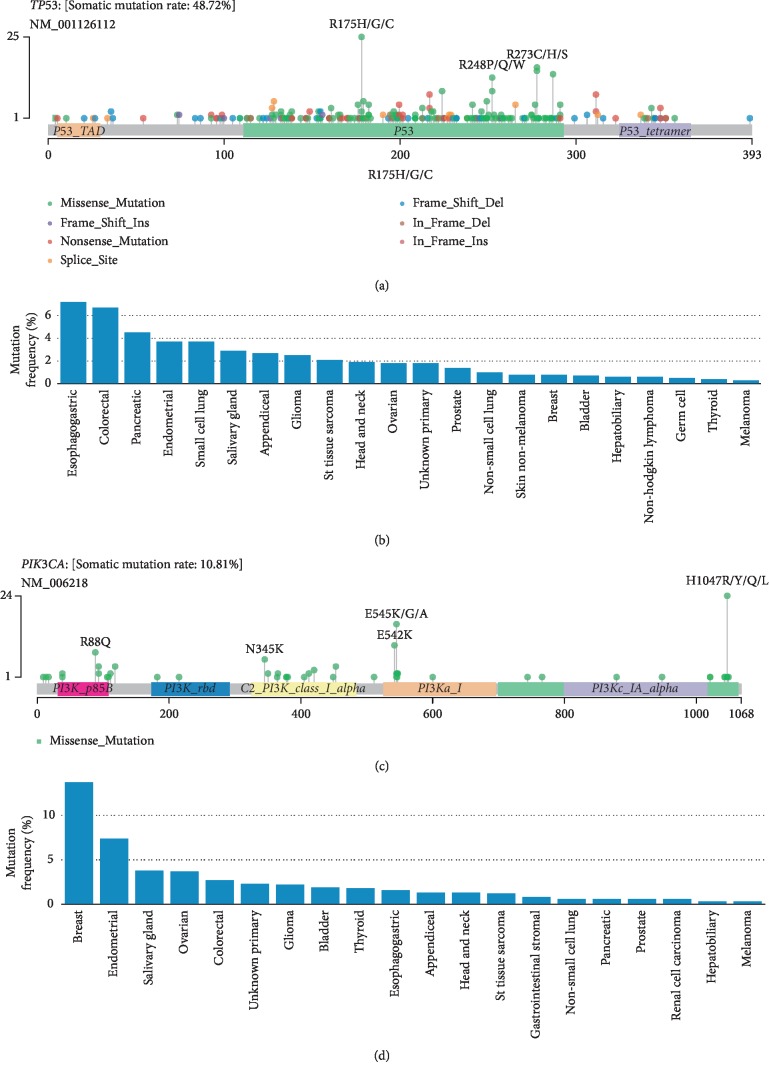
*TP53* mutational spectrum in 942 GC patients (a) and *TP53* R175H mutation in MSK-IMPACT pan-cancer cohorts (b). *PIK3CA* mutational spectrum in 942 GC patients (c) and *PIK3CA* H1047R mutation in MSK-IMPACT pan-cancer cohorts (d).

*TP53* is the most common mutated tumor suppressor gene in GC and other cancers, and *TP53* R175H mutation is known to be carcinogenic [36], which located in the DNA binding domain of TP53 protein (Figure 4(a)). R175H mutation has a series of adverse effects, such as reducing the activation of the *TP53* target, interfering with the activation of wild-type *TP53*, leading to resistance to apoptosis, reducing genomic stability, and promoting tumorigenesis and cell migration [37, 38]. In TCGA pan-cancer cohort (almost primary cancer, *n* = 11,160), the mutation occurred in 162 (1.5%) individuals. Similarly, the mutation is a hotspot in metastatic cancers. In the MSK-IMPACT study of over 10, 000 metastatic cancers [39], the mutation occurred more than 2% in nine cancers and 7.2% in esophagogastric cancers (Figure 4(b)). Although R175H is widespread in many cancers, there is no targeted drug for this mutation. Therefore, we believe that the mutation may be a potential target for T cell immunotherapy across different cancer types.

*PIK3CA* mutations are mainly found in EBV-positive GC [16], and it is one of the driver genes in cancer. *PIK3CA* H1047R is a gain-of-function mutation located in the kinase region (Figure 4(c)), and it is oncogenic and most common in breast cancer [40, 41]. The frequency of this mutation in TCGA pan-cancer was as high as 298 (2.7%), and it was more than 2% in multiple metastatic cancer types in the MSK-IMPACT cohort (Figure 4(d)). *PIK3CA* gene has many mutation forms at this site (including H1047R, H1047Y, and H1047L). Zhou et al. reported that H1047Y mutation can produce tumor-specific neoantigen in a GC patient [21]. However, it is worth noting that H1047R mutation is the main mutation form of *PIK3CA*, whether in TCGA GC cohort or MSK pan-cancer cohort. For breast cancer patients carrying the mutation, there are targeted drugs for the mutation, such as Alpelisib and Fulvestrant [42]. As mentioned earlier, *PIK3CA* mutation and *TP53* mutation are exclusive in GC, so for *TP53* mutation negative patients, T cell immunotherapy based on *PIK3CA* H1047R may be an option, and it is also a potential choice for breast cancer or other cancer types that are resistant to targeted drugs. In addition, *KRAS* Gly12 (including G12V, G12C, and G12D) is also a classic cancer mutation, and the mutation frequency of this site in metastatic pancreatic and appendiceal cancers is more than 20% [39]. Both Charoentong and Witkiewicz have reported the high frequency of the *KRAS* G12D mutation in pancreatic cancer [20, 43]. In fact, the first clinical trial to test this immunotherapy regimen in HLA-A11:01 cancer patients with *KRAS* G12V mutation has begun (NCT03190941).

## 4. Discussion

Before this study, Zhou et al. had published a neoantigen prediction study for GC. However, there are some limitations in their research, which have been solved in this study. Firstly, their study only involved 32 samples of GC, and no neoantigens were found shared among GC patients. Secondly, they only used a neoantigen prediction software, several neoantigen prediction software have been released and updated recently [29–33], and research shows that the combination of multiple software can better improve the accuracy and sensitivity of neoantigen prediction [32]. Thirdly, we found several neoantigens associated mutation significantly mutated in GC patients or pan-cancer cohort, such as *PIK3CA* H1047R and *TP53* R175H, which were not found in previous studies. Finally, mutants of *MUC4* were reported in 94% (30/32) of patients in the Zhejiang cohort, but previous studies in several GC cohorts have never found such a high mutation frequency of the gene *MUC4* [14–19, 34, 35]. It seems that their study has obvious false-positive results in mutation detection, and the accuracy of mutation detection results will directly affect the predictive results of neoantigens.

For the recurrent neoantigens identified in this study, there are a variety of HLA alleles that can present them to the cell surface (Table 2). Among them, HLA-A11:01, HLA-A24:02, HLA-A02:01, and HLA-C07:02 mainly appeared in the Chinese population [44], indicating that cancer patients with these HLA-type in China will be potential therapeutic populations for these “public” neoantigens. Of course, there is still a long way to go for the immunotherapy of these “public” neoantigens. Firstly, these neoantigens should be experimentally confirmed to be presented and recognized by T cells. Secondly, it is necessary to ensure that normal tissue cells do not express these antigens in order to avoid the adverse autoimmune reactions of T cells attacking normal tissues. These can be accomplished with the help of highly sensitive mass spectrometry-based assay and a series of experiments [45].

In this study, in order to promote the immunotherapy of GC based on neoantigens, we combined 74 samples of Fujian cohort with published sequencing data to obtain 942 samples and carried out the largest study of the mutation and neoantigen landscape of GC so far. Firstly, we constructed the most complete mutational profiling of GC so far. Notably, we found five genes were mutated exclusively with *TP53*, including *CDH1*, *KMT2D*, *RYR1*, *PIK3CA*, and *ARID1A* (Fisher's exact test, *P* < 0.05, [Supplementary-material supplementary-material-1]), which four of them (*CDH1*, *KMT2D*, *RYR1*, and *PIK3CA*) were not reported before. Secondly, we use a combination of multiple software to detect neoantigens, avoiding the limitations of a single software (for example, PSSMHCpan [32] can pan-specifically predict peptides that bind to 4896 HLA class I alleles, while NetMHC-4.0 [29] and NetMHCpan-3.0 [30] can predict only 89 and 2924 HLA class I alleles, respectively) [32]. Thirdly, the purpose of this study is not only to obtain the neoantigens of GC but also to find potential common antigens in GC patients and to promote the immunotherapy of GC. By combining Fujian and TCGA cohorts, 482 GC samples were predicted for neoantigens, and 549 neoantigens were found to be shared among the samples. If we consider the top ten neoantigens as a panel, it can cover about 15.8% of the total 482 samples. At present, 10-20 cancer-specific neoantigens are usually synthesized in vitro and used in T cell immunotherapy [11]. Since these neoantigens can cover a certain proportion of GC population, we believe that these recurrent neoantigens of GC have potential clinical application value. It is believed that, with the development of neoantigen identification technology, more kinds and more quantities of neoantigens will be discovered, such as those produced by tumor-specific expressed abnormal splicing transcripts from noncoding regions of human genome [46], neoantigen-based immunotherapy will play an increasingly important role in cancer treatment.

## 5. Conclusions

Overall, based on 942 whole exome/genome sequencing data of Fujian samples and other published data, the most complete mutation landscape of GC was obtained. Based on the mutation data and HLA information, several recurrent neoantigen-associated mutations, such as *PIK3CA* H1047R and *TP53* R175H, were predicted. Some of these neoantigen-associated mutations also have high frequencies in pan-cancer, indicating that they are potential targets for pan-cancer immunotherapy.

## Figures and Tables

**Table 1:** Summary of clinical information of Fujian cohort, TCGA cohort, and 942 integrated GC samples.

Characteristics	Fujian	TCGA	All
Age			
<60	28	132	318
≧60	46	306	589
NA	0	5	35

Sex			
Male	52	285	607
Female	22	158	315
NA	0	0	20

Lauren's type			
Intestinal	32	191	429
Diffuse	28	72	305
Mixed	10	0	24
NA	4	180	184

Tumor stage			
Stage I	7	59	96
Stage II	7	130	200
Stage III	51	183	418
Stage IV	9	44	178
NA	0	27	50

Differentiation			
Poor	51	—	136
Well	20	—	21
Moderate	0	—	32
NA	3	443	753

Location			
Antrum	17	162	346
Body	31	152	286
Cardia	22	62	175
Others	4	46	59
NA	0	21	76

NA, not available.

**Table 2:** The list of top 10 neoantigens, their corresponding mutation (AA change), neoantigen frequency in Fujian and TCGA cohorts (Freq1, *n* = 482), mutation frequency in all GC cohort (Freq2, *n* = 942), and TCGA pan-cancer cohort (Freq3, *n* = 11,160), and HLA information.

Gene	AA change	Freq1	Freq2	Freq3	HLA types
*PGM5*	I98V	10	26	51	HLA-A02:30; HLA-A02:01; HLA-A02:03; HLA-A02:05; HLA-A02:06; HLA-A02:07; HLA-C03:03
*TP53*	R175H	8	25	162	HLA-C07:02; HLA-C07:01; HLA-A68:01; HLA-A02:03
*TRIM49C*	S327R	7	10	17	HLA-B35:03; HLA-C06:02; HLA-C12:03; HLA-C07:01; HLA-C07:02; HLA-B27:08; HLA-B07:02; HLA-A02:01; HLA-B27:05; HLA-B57:01; HLA-A32:01; HLA-A11:01; HLA-A31:01
*PIK3CA*	H1047R	5	24	298	HLA-C07:02; HLA-C07:01; HLA-A30:01; HLA-B58:01; HLA-B57:01; HLA-A33:03; HLA-A68:01
*TP53*	R273H	5	16	145	HLA-A02:01; HLA-A02:07; HLA-A02:17; HLA-A68:01
*KRAS*	G13D	5	15	78	HLA-A02:01; HLA-A11:01; HLA-A68:01
*ERBB3*	V104M	5	11	33	HLA-C06:02; HLA-B08:01; HLA-C07:01; HLA-A30:01; HLA-C12:03; HLA-B35:01; HLA-A30:02; HLA-A68:01; HLA-B07:02; HLA-A03:01; HLA-C07:02; HLA-A02:01
*C6*	K817T	4	5	9	HLA-C03:04; HLA-C07:01; HLA-C03:03; HLA-C12:02; HLA-A02:01; HLA-C02:10; HLA-A24:02; HLA-B35:01; HLA-B15:03; HLA-A31:01
*KRAS*	G12D	4	16	430	HLA-A02:01; HLA-C05:01; HLA-A02:06; HLA-A11:01; HLA-A03:01; HLA-B07:02
*TP53*	R282W	4	14	97	HLA-C03:03; HLA-A11:01; HLA-A03:01; HLA-B07:02; HLA-A68:01
*OR4C16*	S135R	4	6	8	HLA-B37:01; HLA-A02:06; HLA-A02:01; HLA-B15:01; HLA-C03:03; HLA-B27:05; HLA-A03:01; HLA-C07:02; HLA-A24:02

## Data Availability

The data reported in this study are available in the CNGB Nucleotide Sequence Archive (CNSA: https://db.cngb.org/cnsa;accession number CNP0000159). Other data can be obtained by contacting the corresponding author.

## Supplementary Materials

Supplementary Materials Supplementary Figure S1: mutational landscape of the 942 GC (gastric cancer) samples. Supplementary Figure S2: somatic mutation interactions between genes. Supplementary Table S1: six published GC genomic literature involving 942 GC samples in this study. Supplementary Table S2: clinical information of 942 GC samples. Supplementary Table S3: mutation sites, frequencies, and HLA I alleles of all predicted neoantigens.
