# Supplementary figures and images for: A Comprehensive Survey of Genomic Alterations in Gastric Cancer Reveals Recurrent Neoantigens as Potential Therapeutic Targets
Source: Biomed Res Int. 2019 Nov 5;2019:2183510. doi: 10.1155/2019/2183510 (PMC6874998; doi:10.1155/2019/2183510)

Altered in 842 (90.15%) of 934 samples.

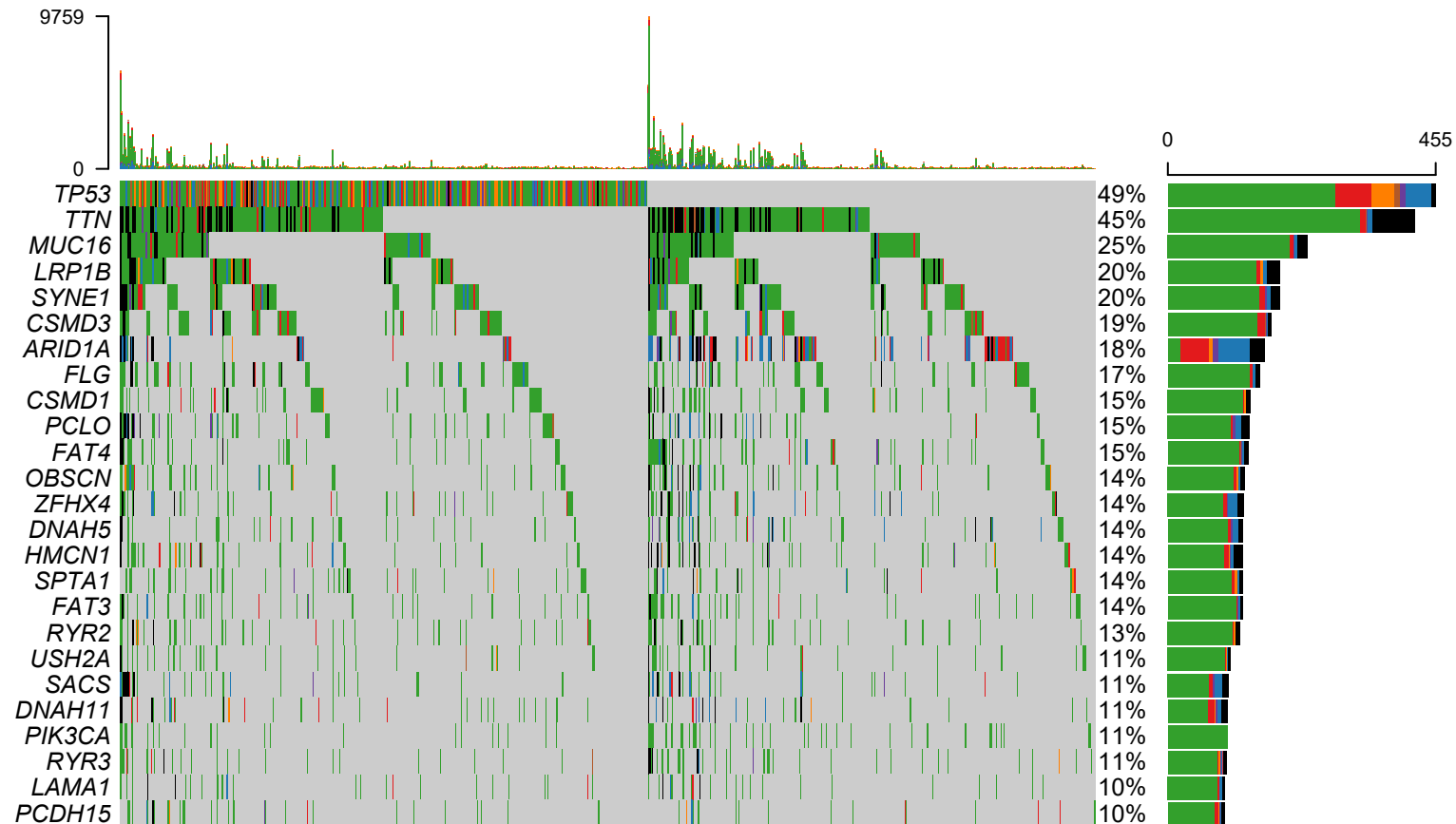

Supplement: Supplementary Materials — Supplementary Figure S1: mutational landscape of the 942 GC (gastric cancer) samples. Supplementary Figure S2: somatic mutation interactions between genes. Supplementary Table S1: six published GC genomic literature involving 942 GC samples in this study. Supplementary Table S2: clinical information of 942 GC samples. Supplementary Table S3: mutation sites, frequencies, and HLA I alleles of all predicted neoantigens. [file 2183510.f1.zip › 2183510.f1/Supplementary Figure S1.pdf]

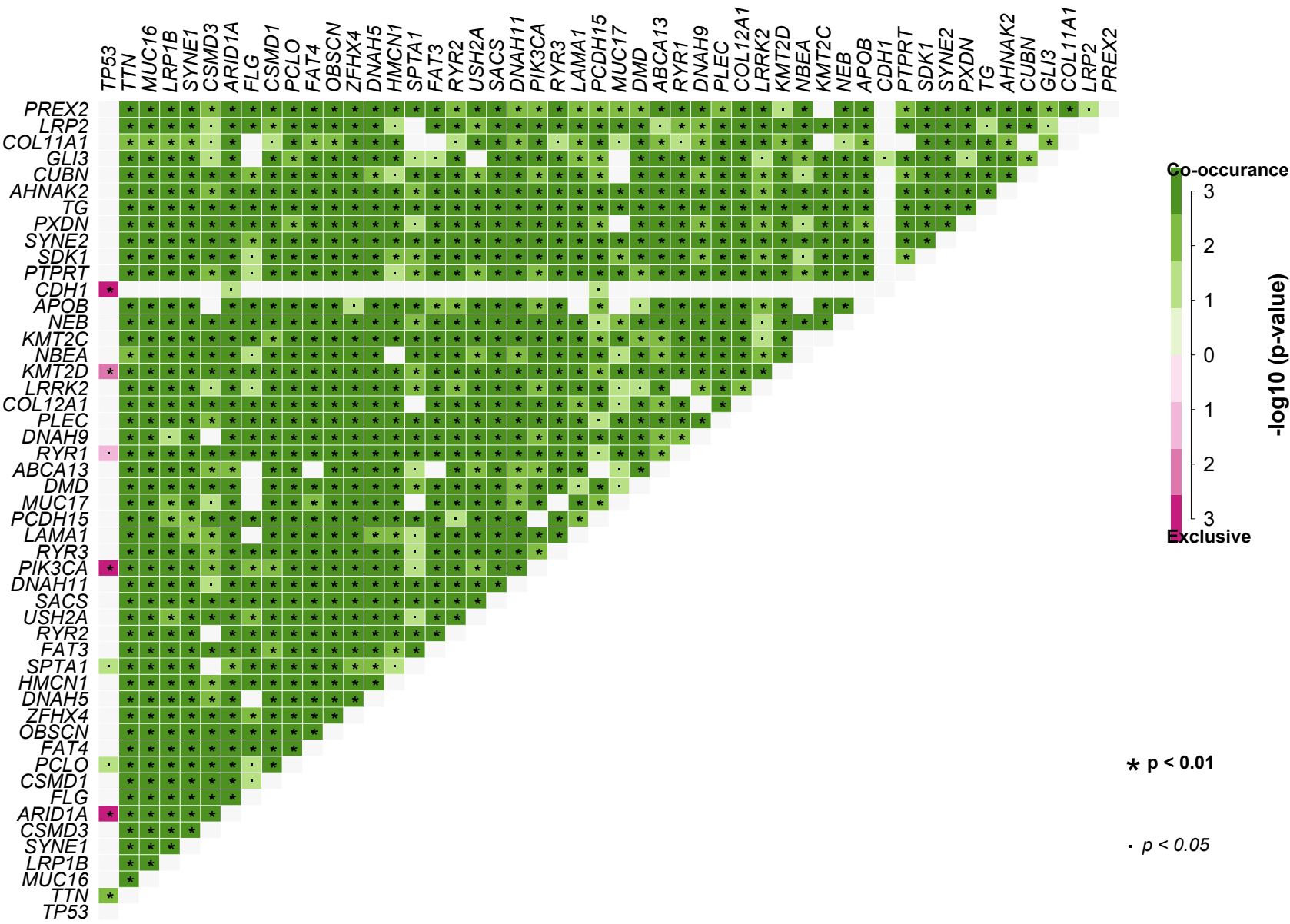

Supplement: Supplementary Materials — Supplementary Figure S1: mutational landscape of the 942 GC (gastric cancer) samples. Supplementary Figure S2: somatic mutation interactions between genes. Supplementary Table S1: six published GC genomic literature involving 942 GC samples in this study. Supplementary Table S2: clinical information of 942 GC samples. Supplementary Table S3: mutation sites, frequencies, and HLA I alleles of all predicted neoantigens. [file 2183510.f1.zip › 2183510.f1/Supplementary Figure S2.pdf]
